# Supplementary material for: Mutant SF3B1 promotes malignancy in PDAC
Source: eLife. 2023 Oct 12;12:e80683. doi: 10.7554/eLife.80683 (PMC10629822; doi:10.7554/eLife.80683)
Supplement: Source data 1. [file elife-80683-data1.zip › Raw Data Gel Images/Raw gel data all.pdf]

Mutant SF3B1 promotes PDAC malignancy through TGF-β resistance

Uncropped agarose gel and western blot images

Related to Fig. 4E: Map3k7 isoform RT-PCR

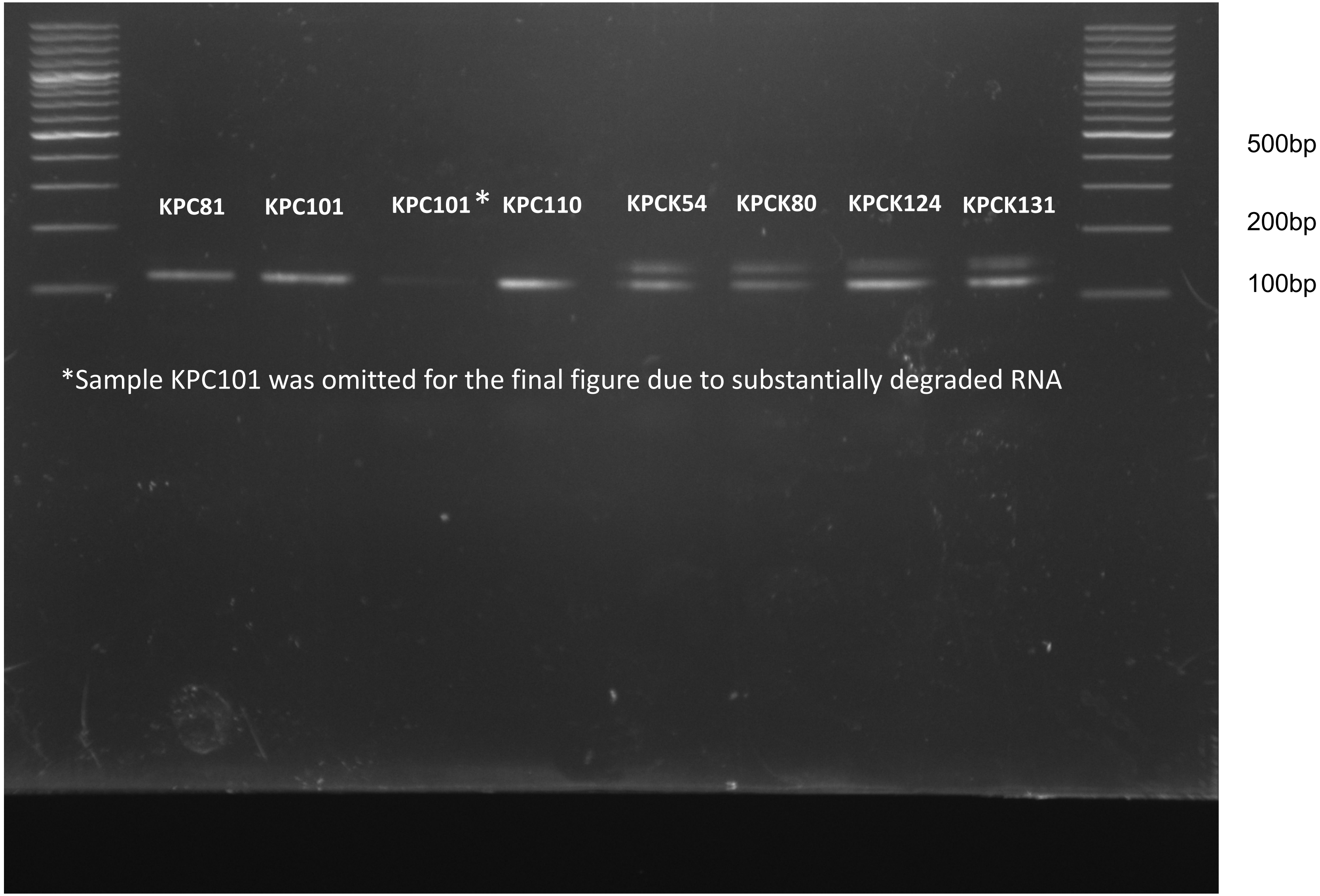

Map3k7 canonical isoform: 114 bp
Map3k7 non-canonical isoform: 137 bp

GeneRuler 100 bp Plus DNA Ladder

Related to Fig. 4F: MAP3K7 isoform RT-PCR

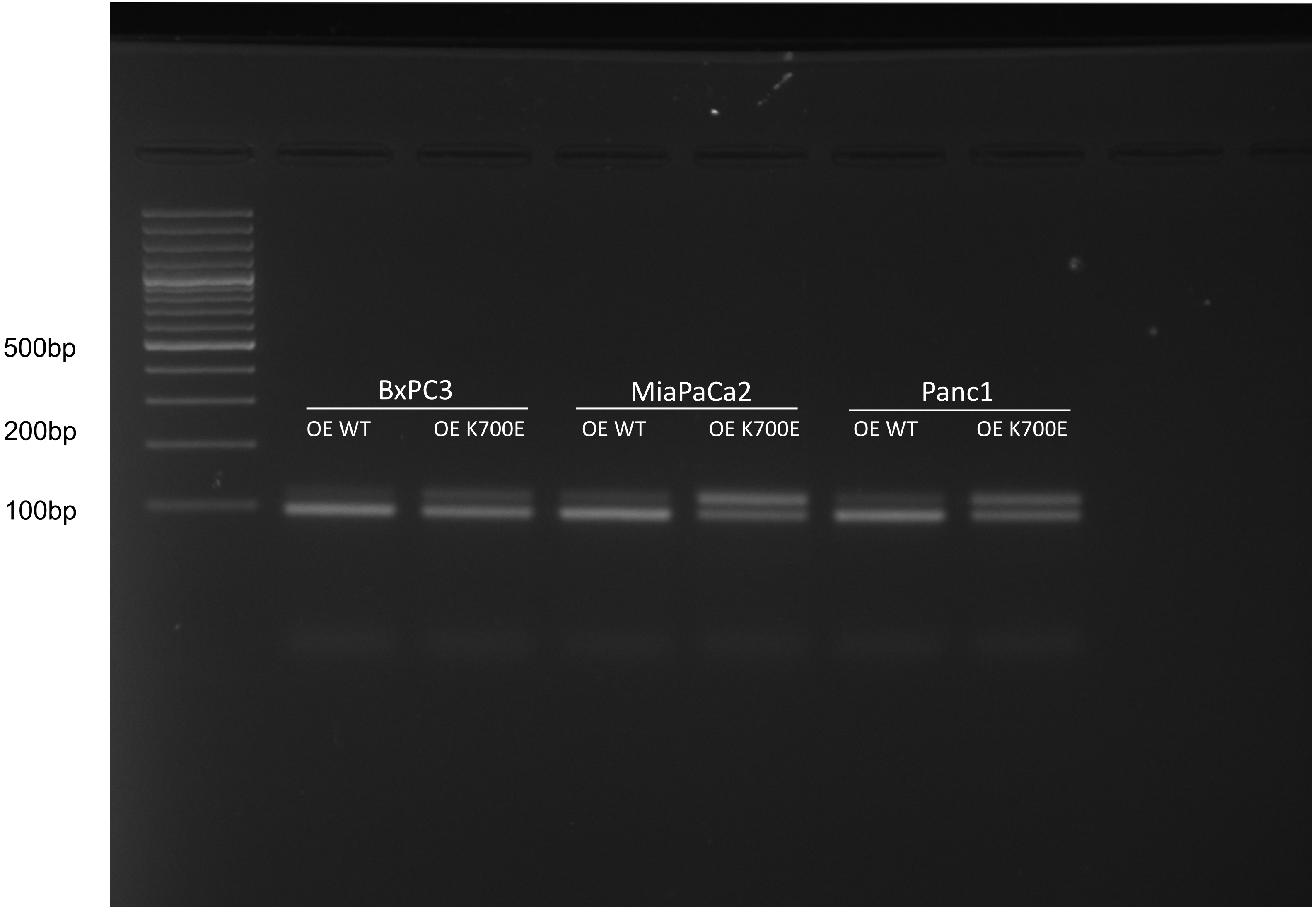

MAP3K7 canonical isoform: 94 bp
MAP3K7 non-canonical isoform: 114 bp

GeneRuler 100 bp Plus DNA Ladder

Related to Fig. 4F: Map3k7 isoform RT-PCR

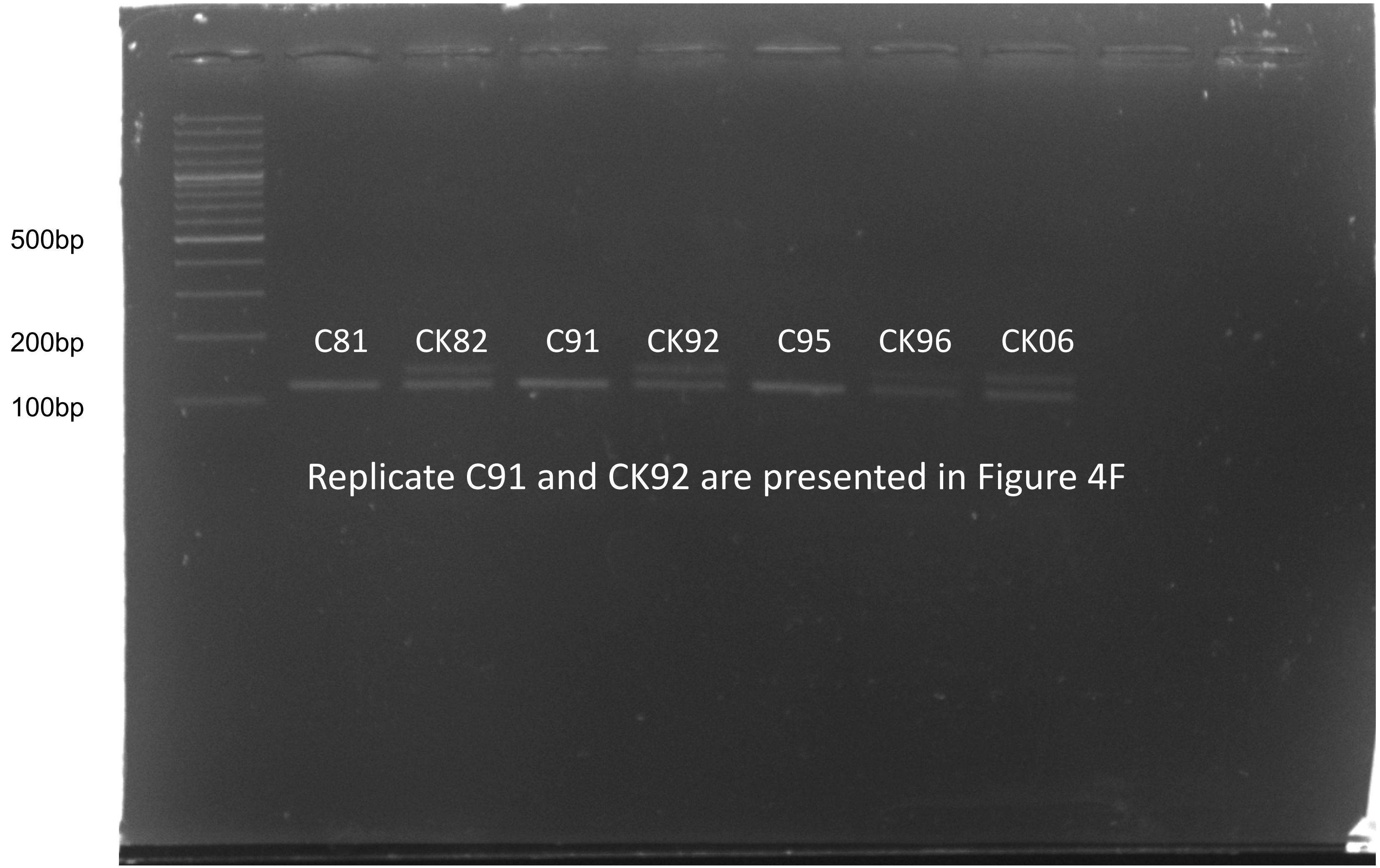

Map3k7 canonical isoform: 114 bp
Map3k7 non-canonical isoform: 137 bp

GeneRuler 100 bp Plus DNA Ladder

Related to Fig. 4E: MAP3K7 isoform RT-PCR

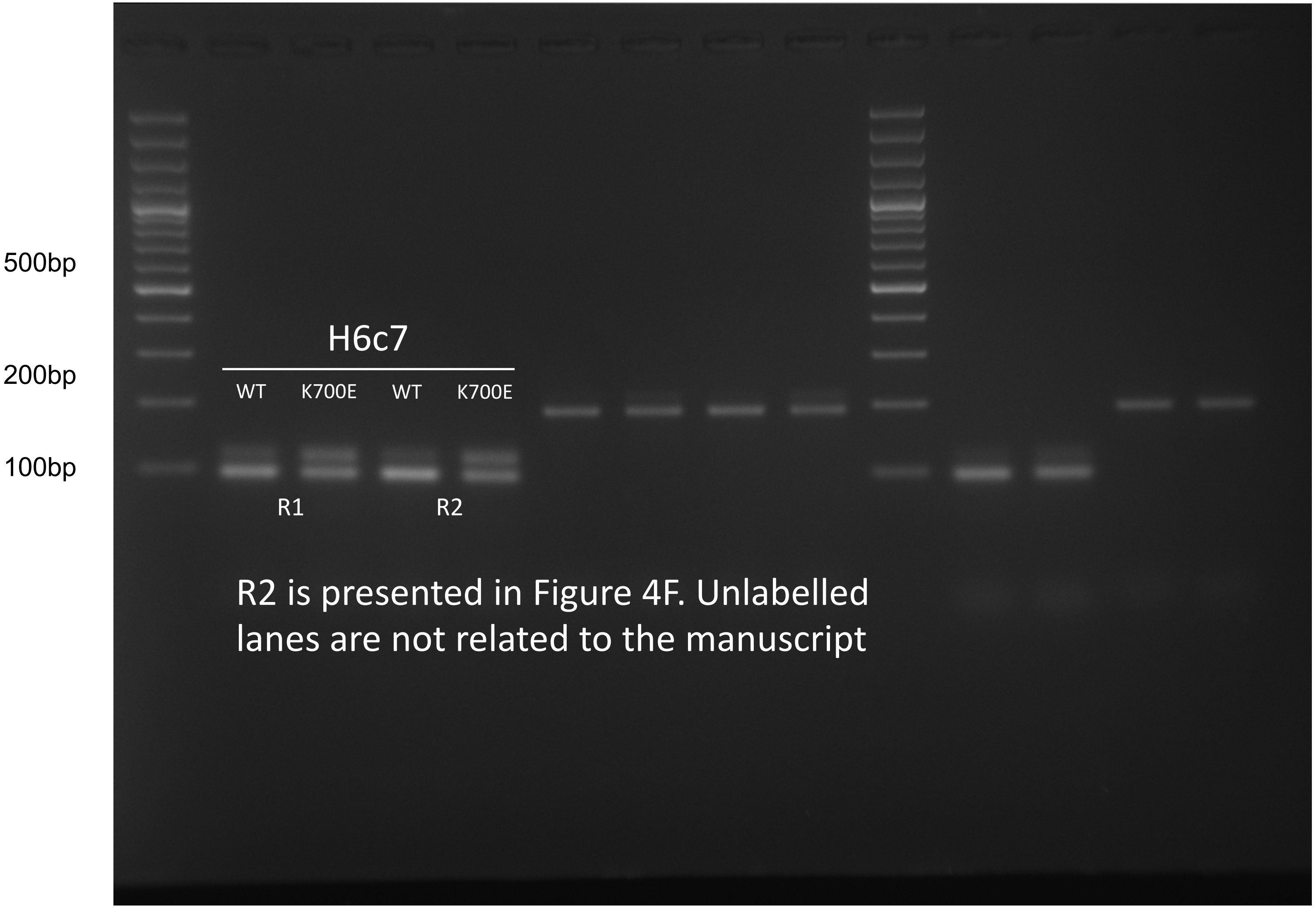

Map3k7 canonical isoform: 94 bp
Map3k7 non-canonical isoform: 114 bp

GeneRuler 100 bp Plus DNA Ladder

Related to Fig. 4E: MAP3K7 isoform RT-PCR

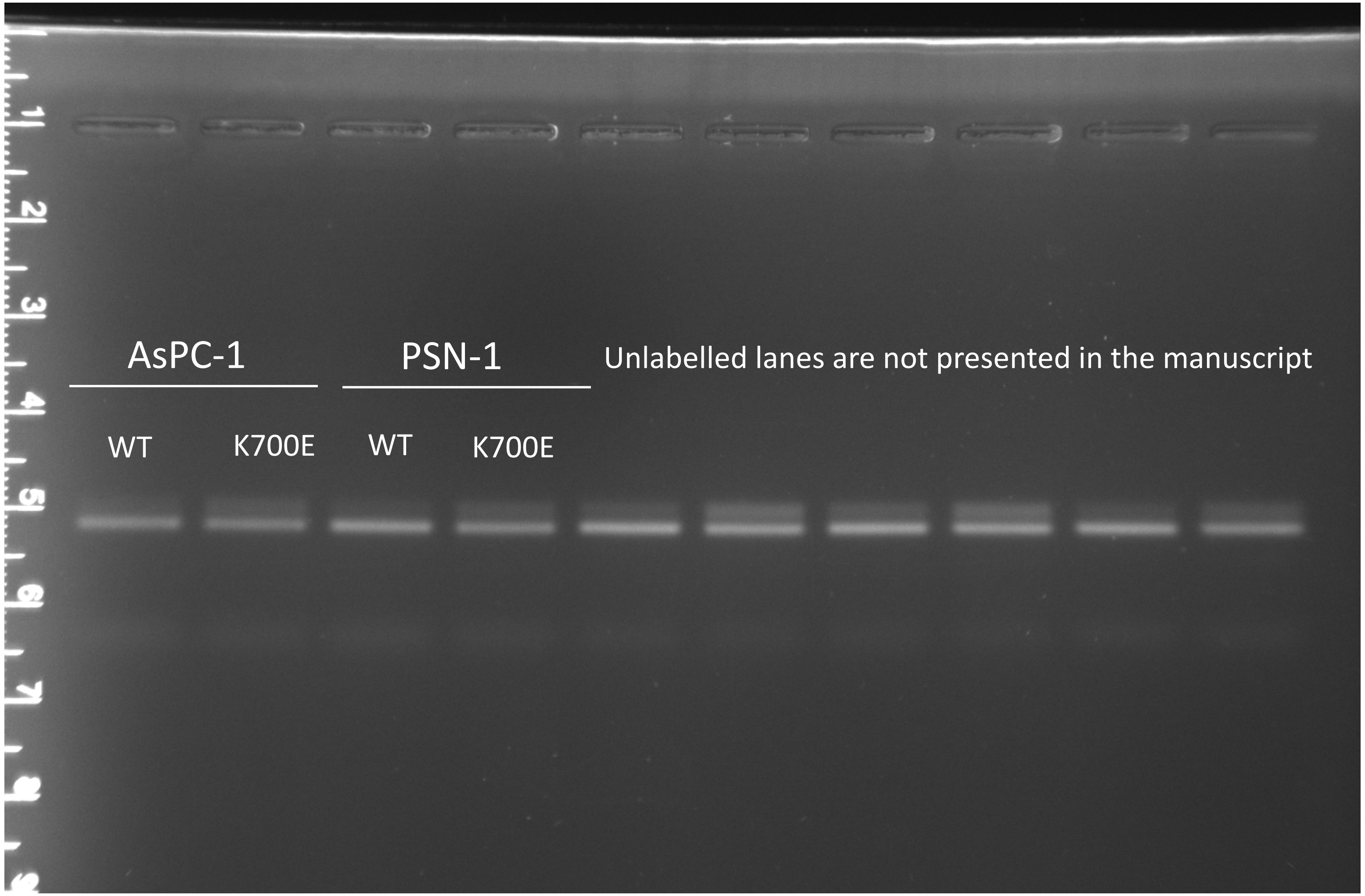

Map3k7 canonical isoform: 94 bp
Map3k7 non-canonical isoform: 114 bp

Related to Suppl. Fig. 4F: Ppp2r5a isoform RT-PCR

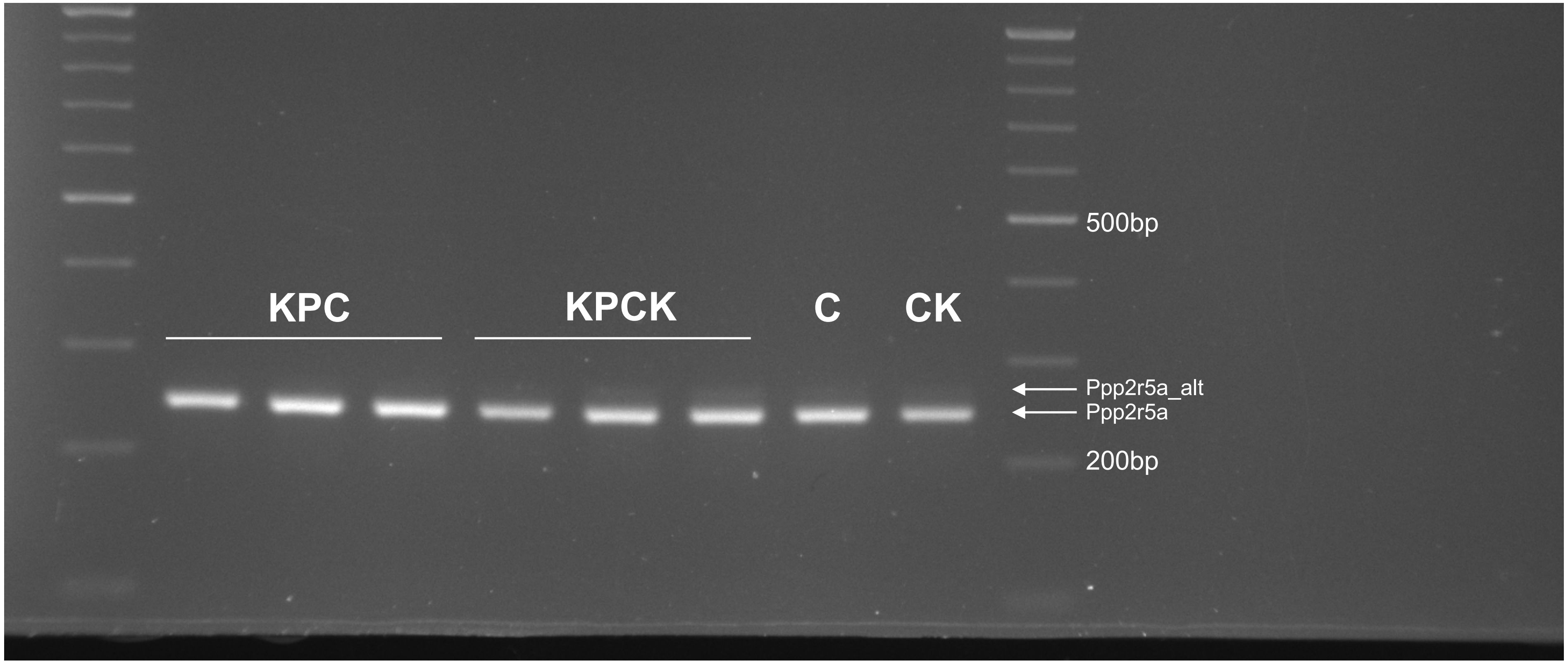

GeneRuler 100 bp Plus DNA Ladder

Related to Suppl. Fig. 4G: Western Blot for MAP3K7

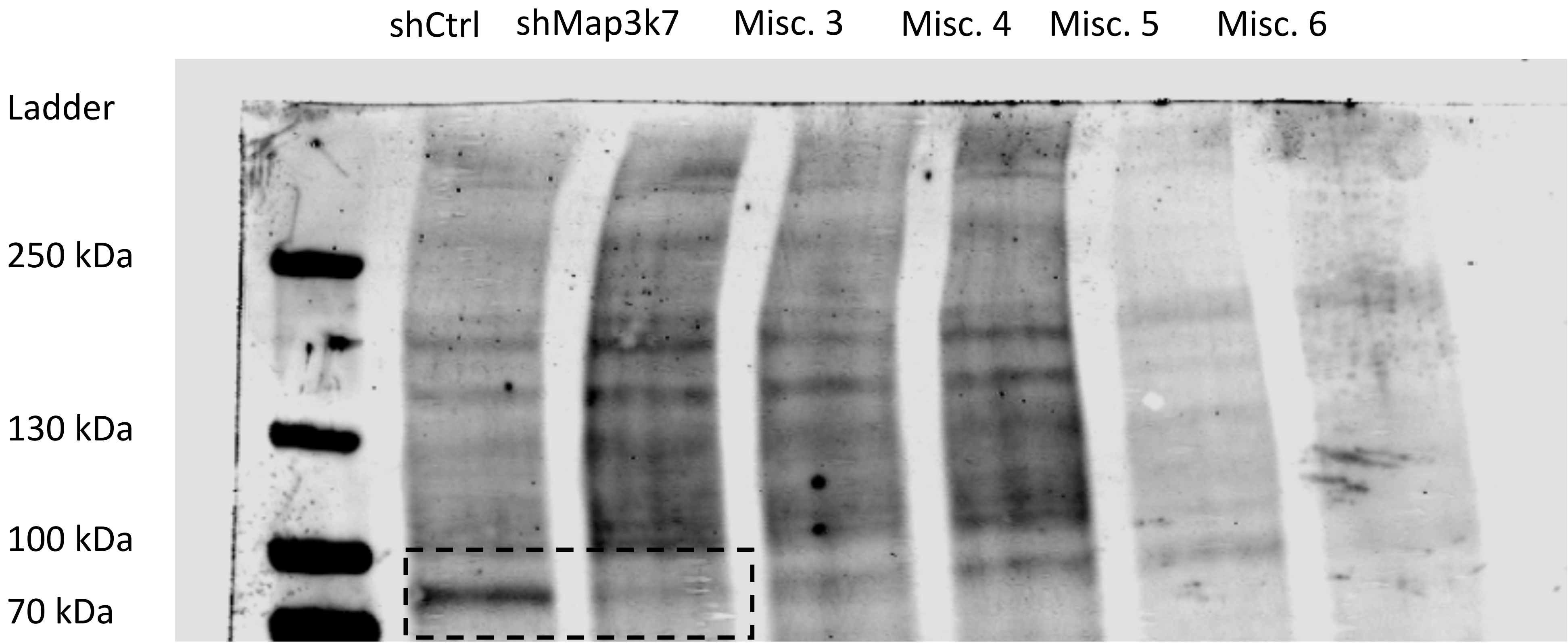

Expected Molecular Weight MAP3K7: 78 to 82 kDa

Dotted line indicates cropped gel image presented in the manuscript
Misc. 3-6 are samples not related to the manuscript

Related to Suppl. Fig. 4G: Western Blot for GAPDH

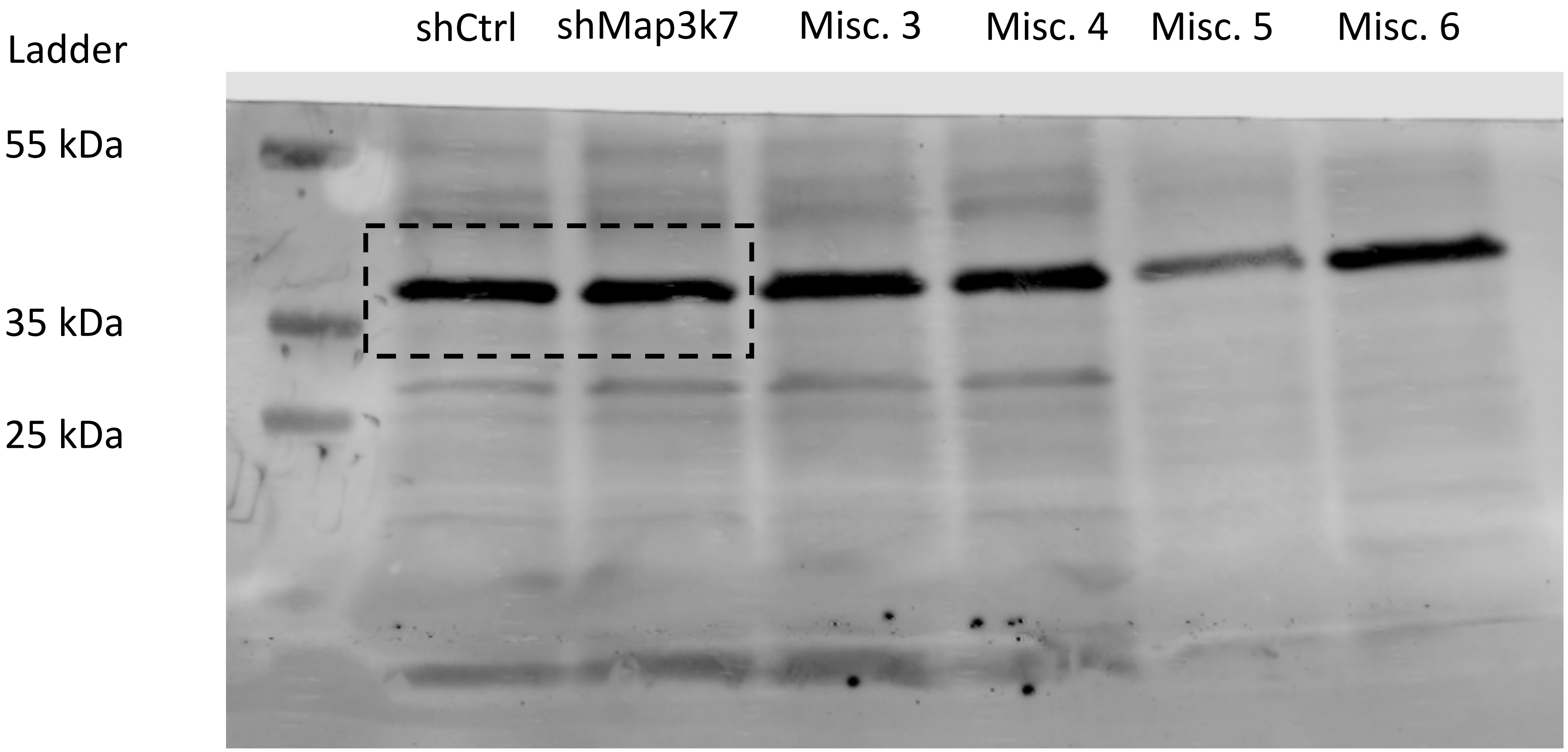

Expected Molecular Weight GAPDH: 36 kDa

Dotted line indicates cropped gel image presented in the manuscript
Misc. 3-6 are samples not related to the manuscript

Related to Fig. 5D: Western Blot for MAP3K7 and GAPDH (short exposure)

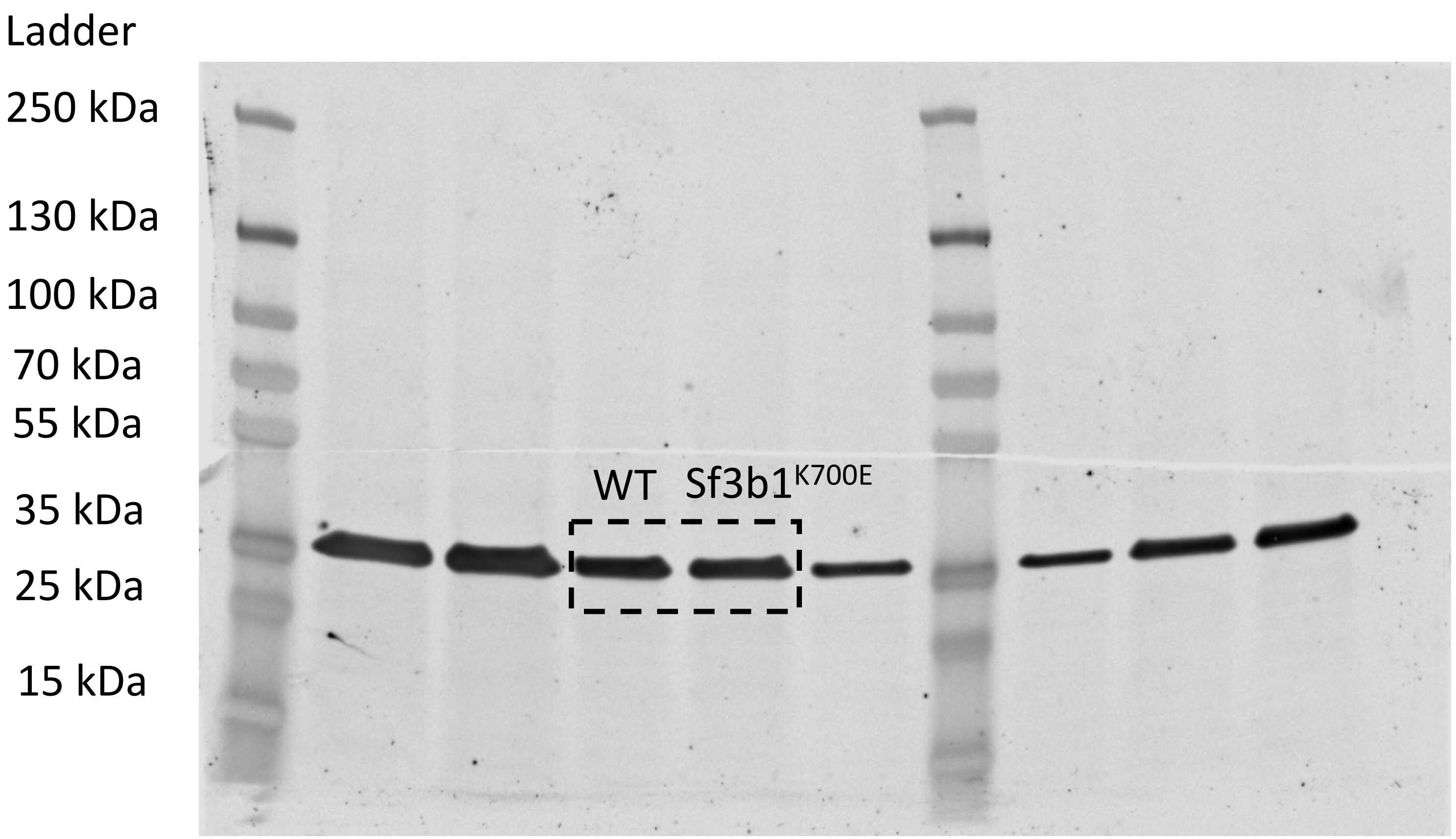

Expected Molecular Weight GAPDH: 36 kDa

Dotted line indicates cropped gel image presented in the manuscript
Unlabelled samples are not presented in the manuscript

Related to Fig. 5D: Western Blot for MAP3K7 and GAPDH (long exposure)

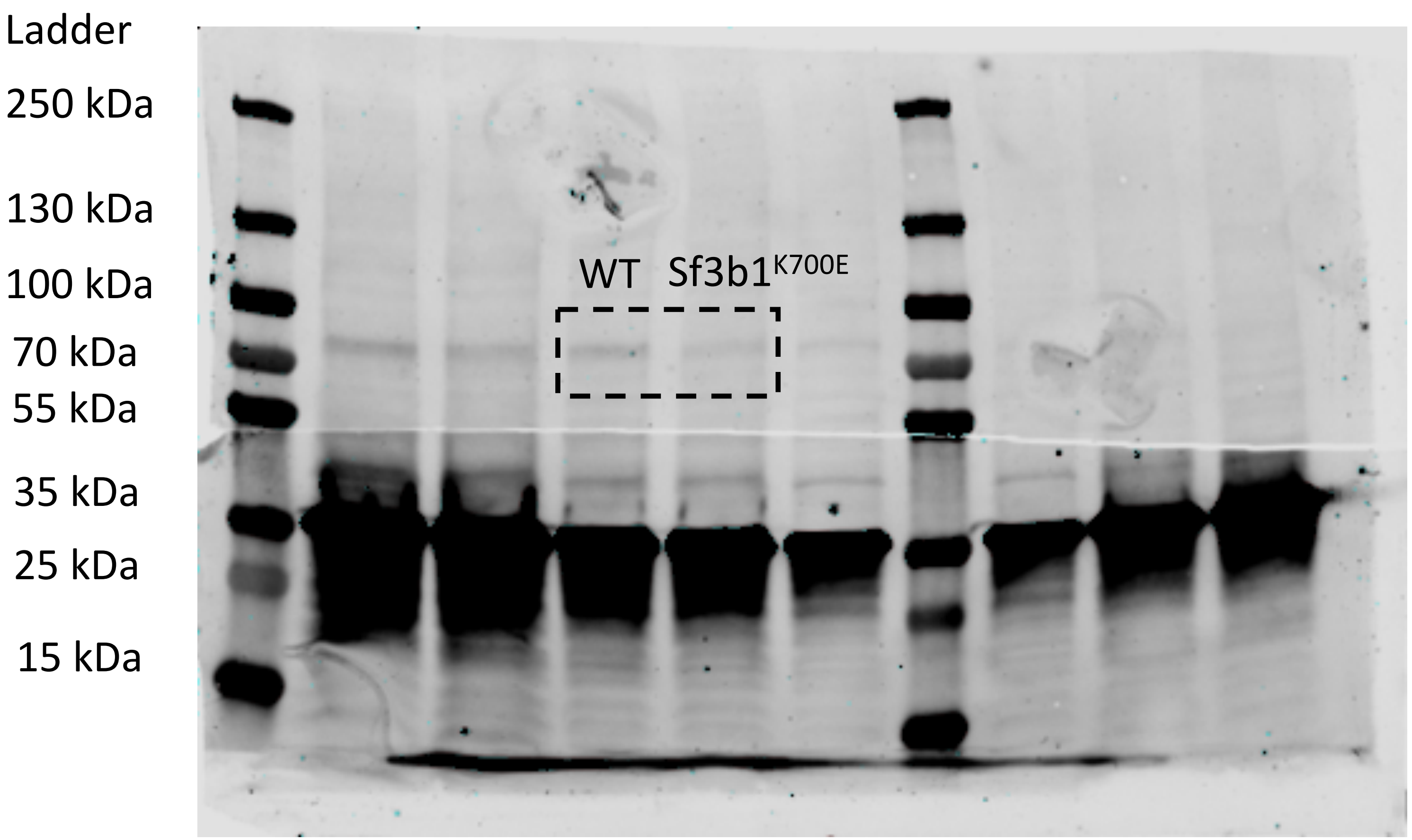

Expected Molecular Weight GAPDH: 36 kDa; MAP3K7: 78-82 kDa

Dotted line indicates cropped gel image presented in the manuscript
Unlabelled samples are not presented in the manuscript

Related to Fig. 5D: Western Blot for MAP3K7

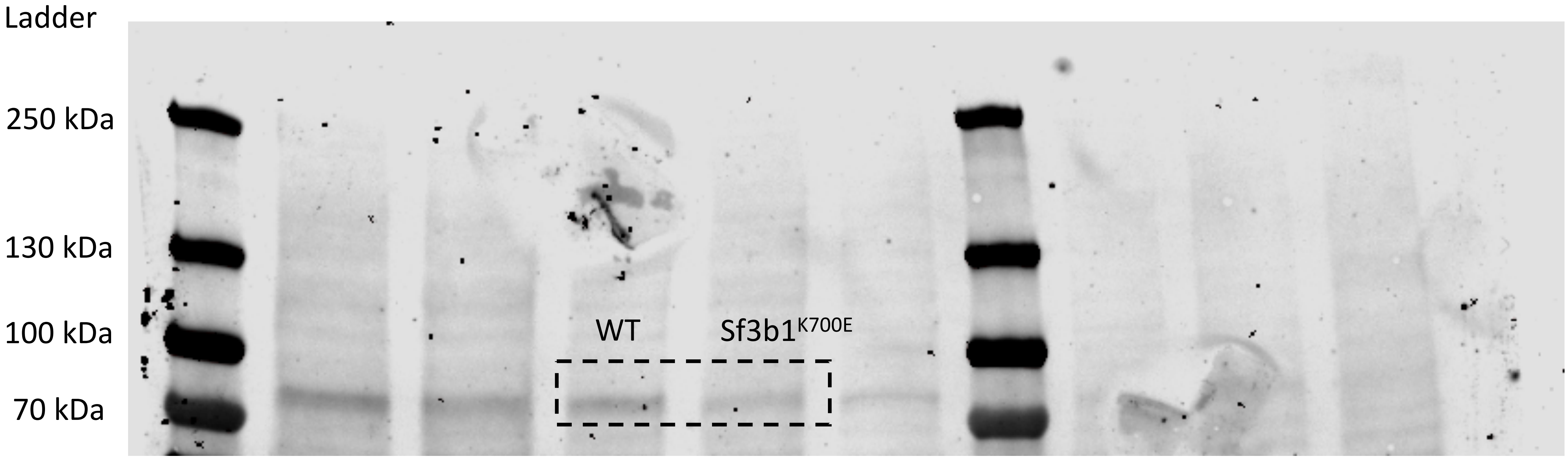

Expected Molecular Weight MAP3K7: 78 to 82 kDa

Dotted line indicates cropped gel image presented in the manuscript
Unlabelled samples are not presented in the manuscript
