# Supplementary figures and images for: Mutant SF3B1 promotes malignancy in PDAC
Source: eLife. 2023 Oct 12;12:e80683. doi: 10.7554/eLife.80683 (PMC10629822; doi:10.7554/eLife.80683)

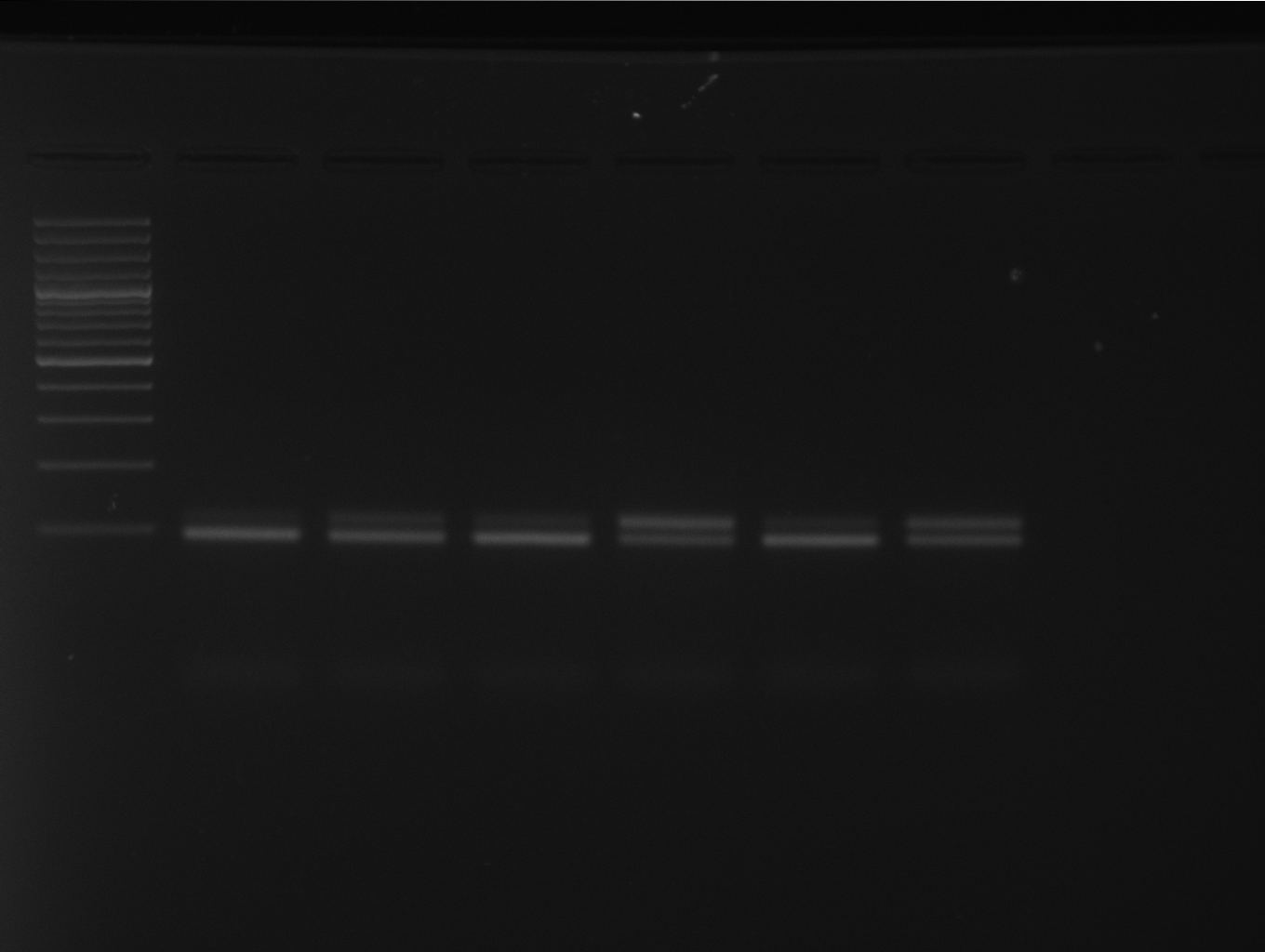

Supplement: Source data 1. [file elife-80683-data1.zip › Raw Data Gel Images/Fig.4F-source-data-1.Tif]

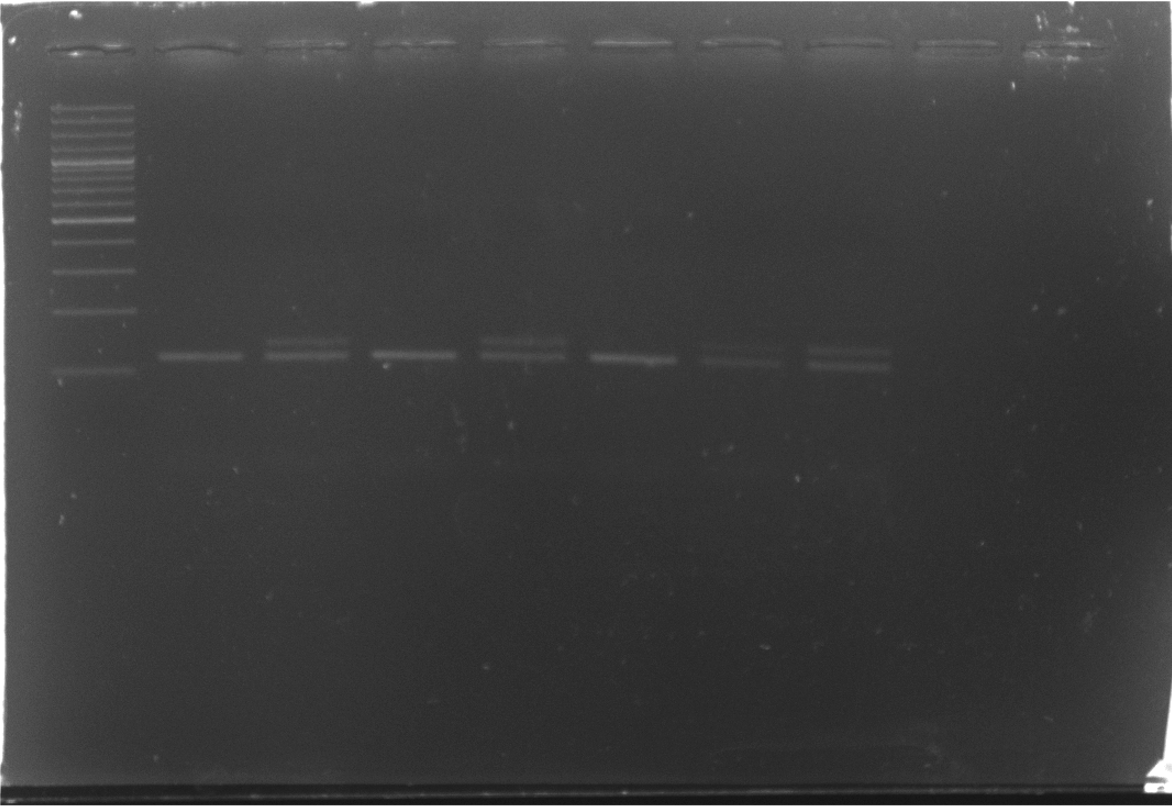

Supplement: Source data 1. [file elife-80683-data1.zip › Raw Data Gel Images/Fig.4F-source-data-2.png]

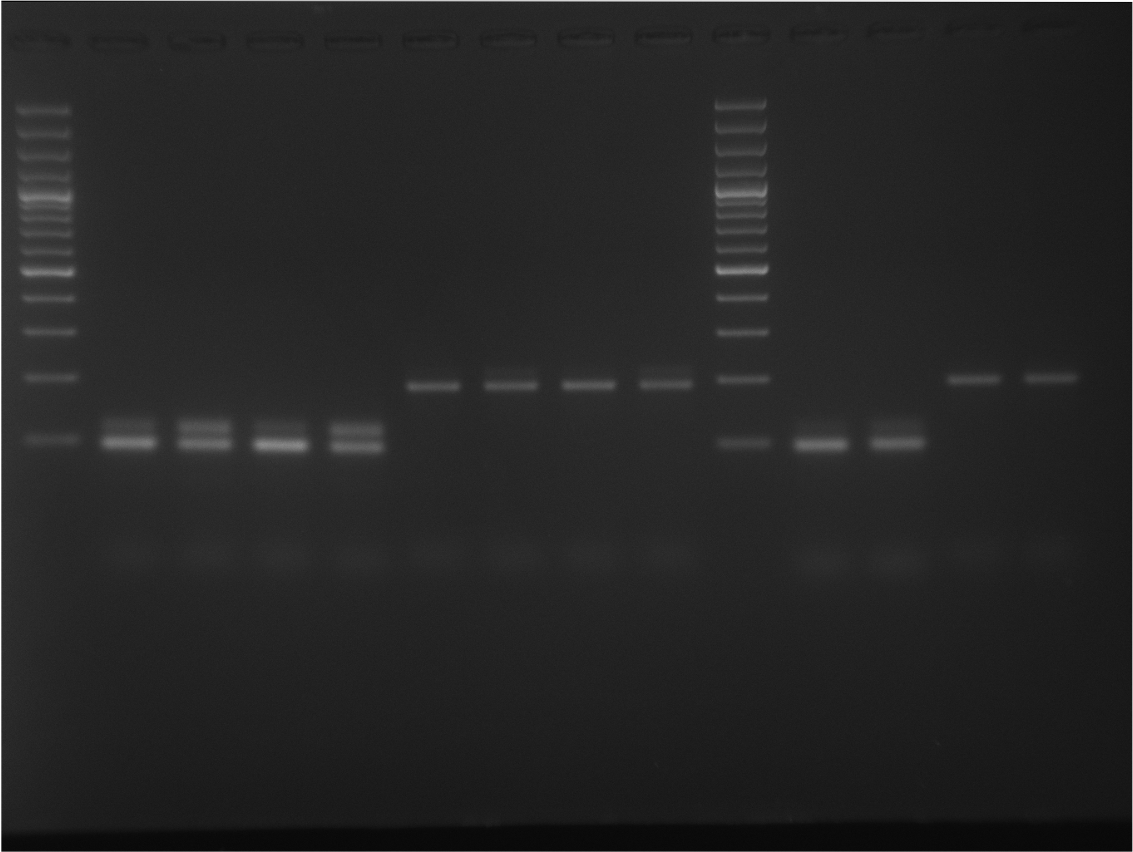

Supplement: Source data 1. [file elife-80683-data1.zip › Raw Data Gel Images/Fig.4F-source-data-3.png]

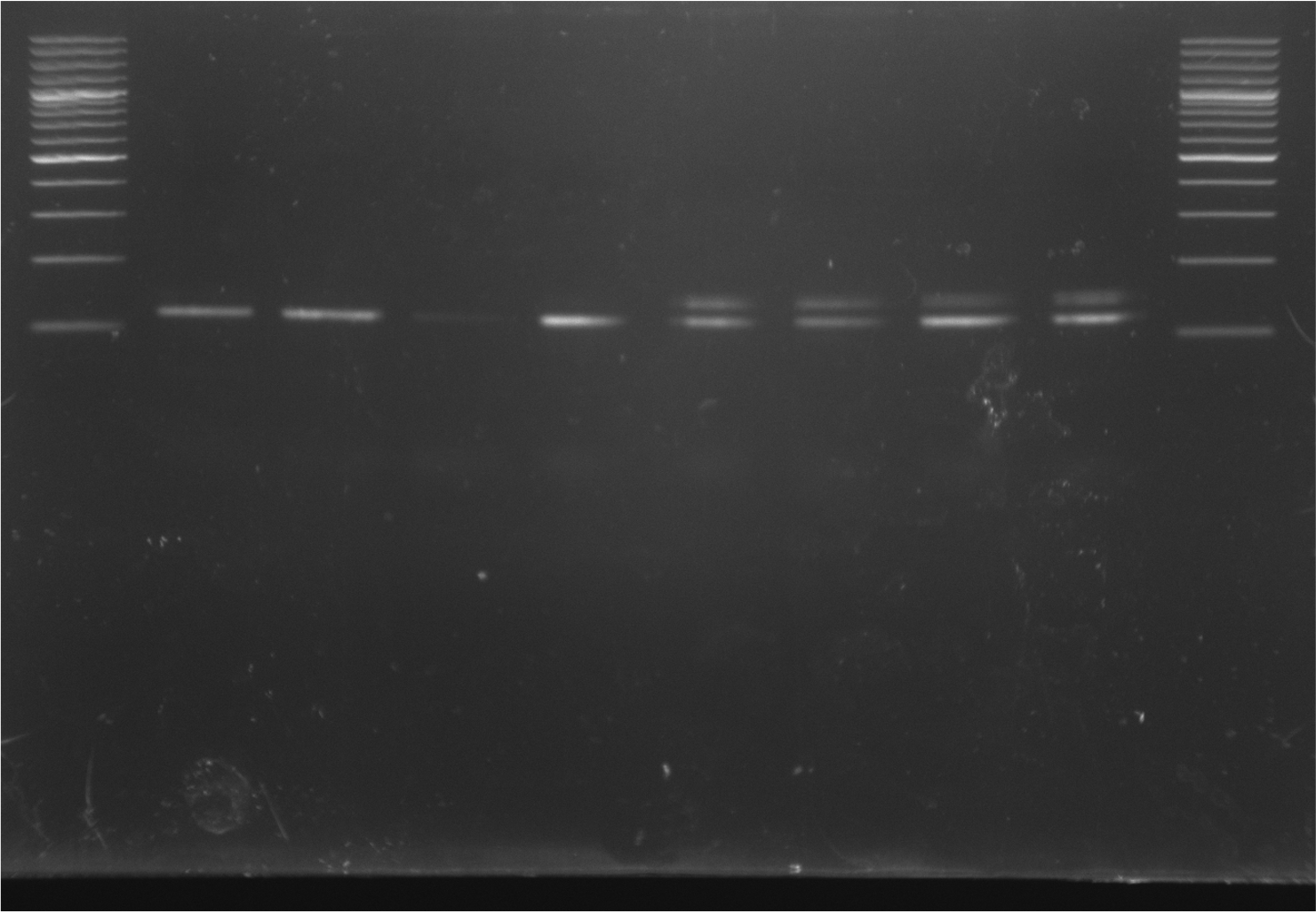

Supplement: Source data 1. [file elife-80683-data1.zip › Raw Data Gel Images/Fig_4E-source-data-1.png]

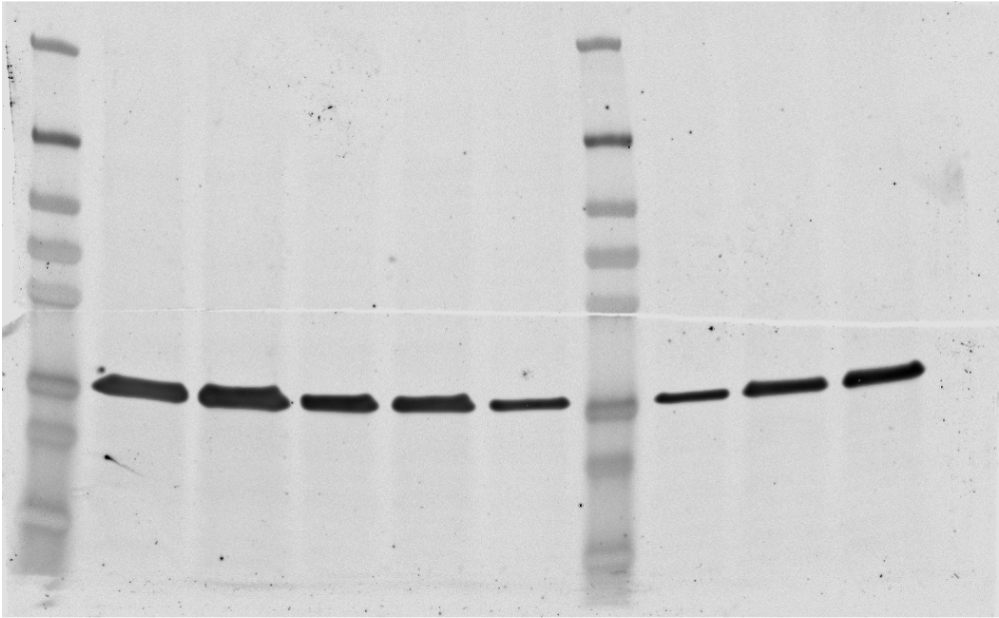

Supplement: Source data 1. [file elife-80683-data1.zip › Raw Data Gel Images/Fig_5D-source-data-1.png]

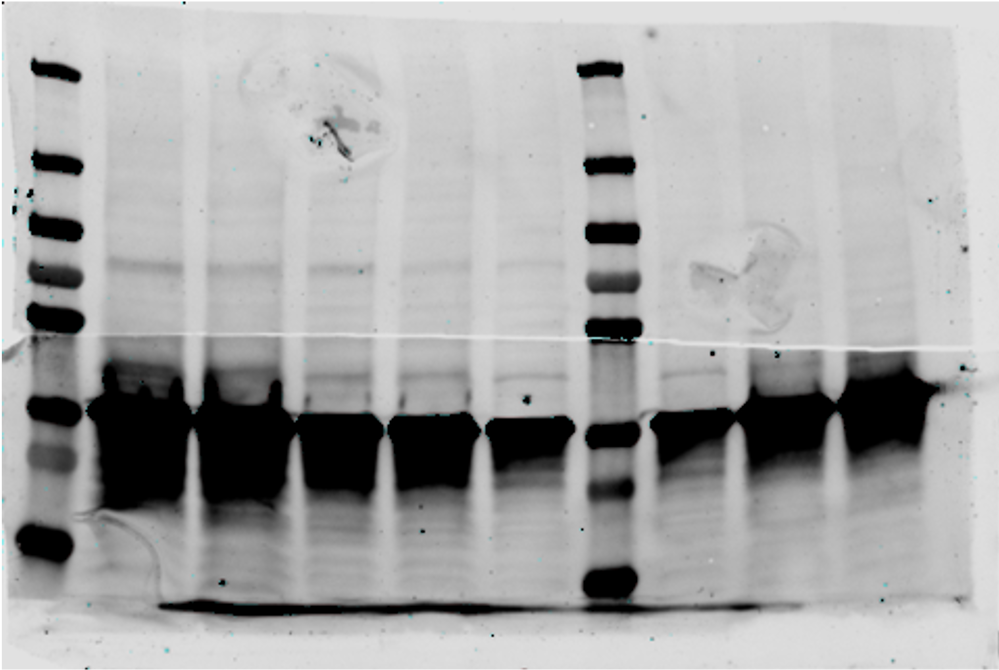

Supplement: Source data 1. [file elife-80683-data1.zip › Raw Data Gel Images/Fig_5D-source-data-2.png]

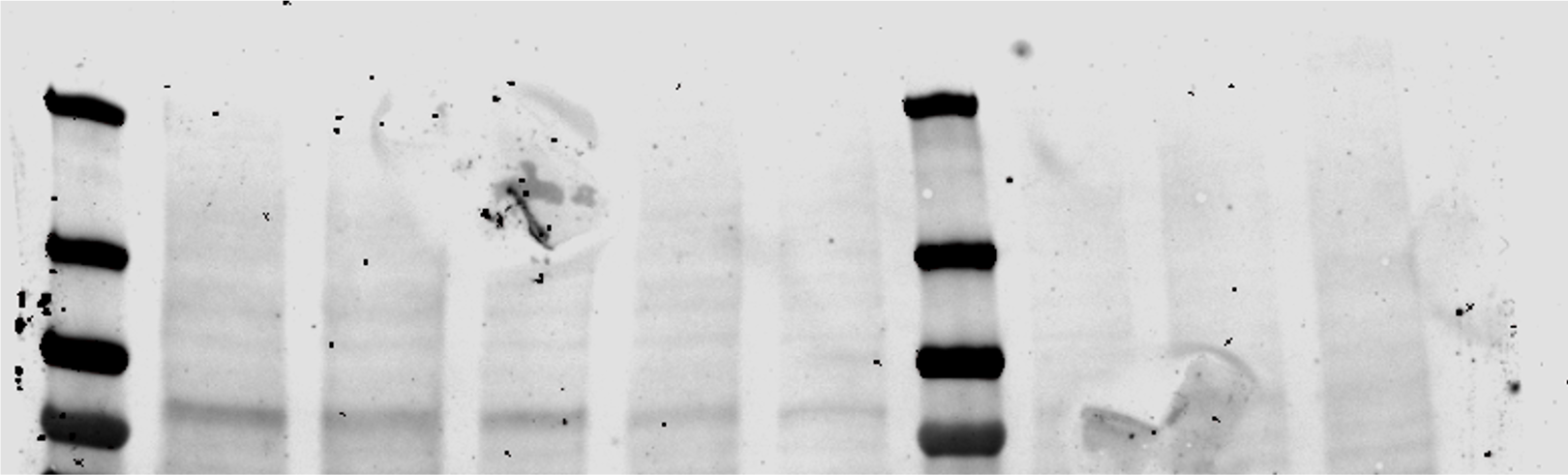

Supplement: Source data 1. [file elife-80683-data1.zip › Raw Data Gel Images/Fig_5D-source-data-3.png]

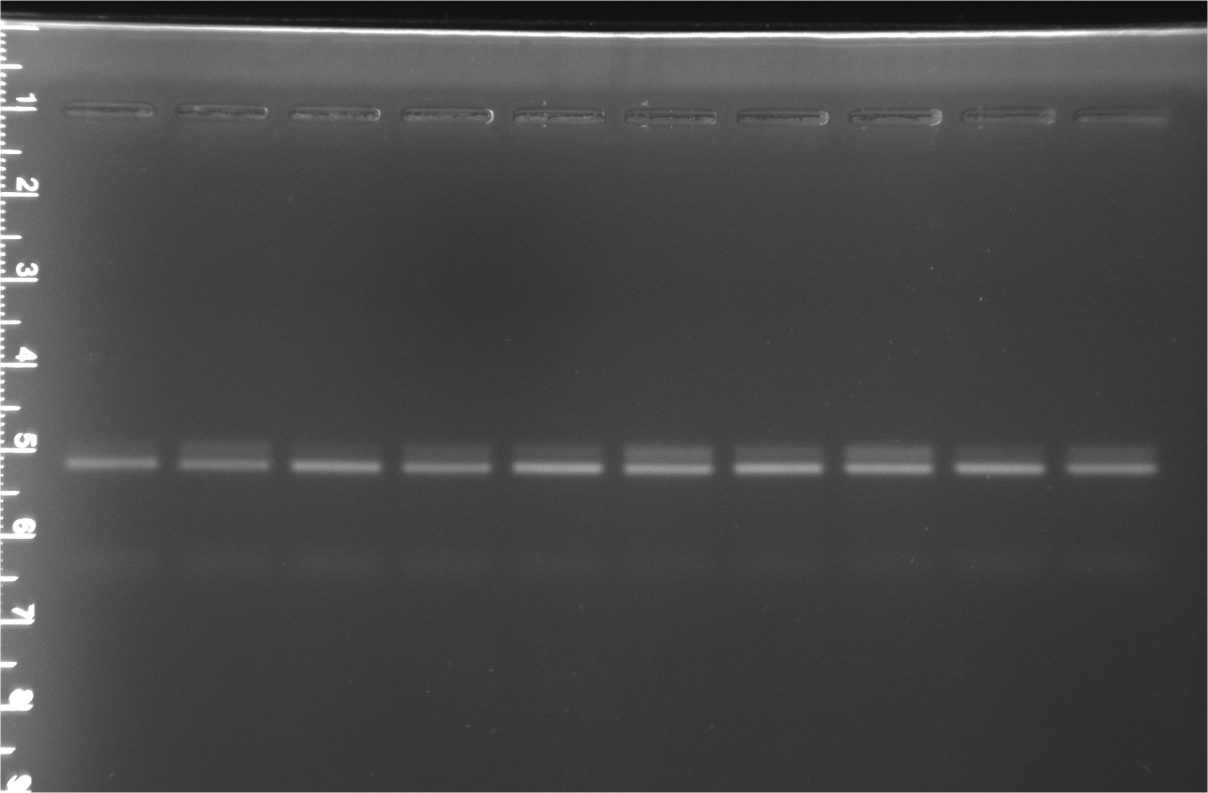

Supplement: Source data 1. [file elife-80683-data1.zip › Raw Data Gel Images/Suppl_Fig_4D-source-data-1.png]

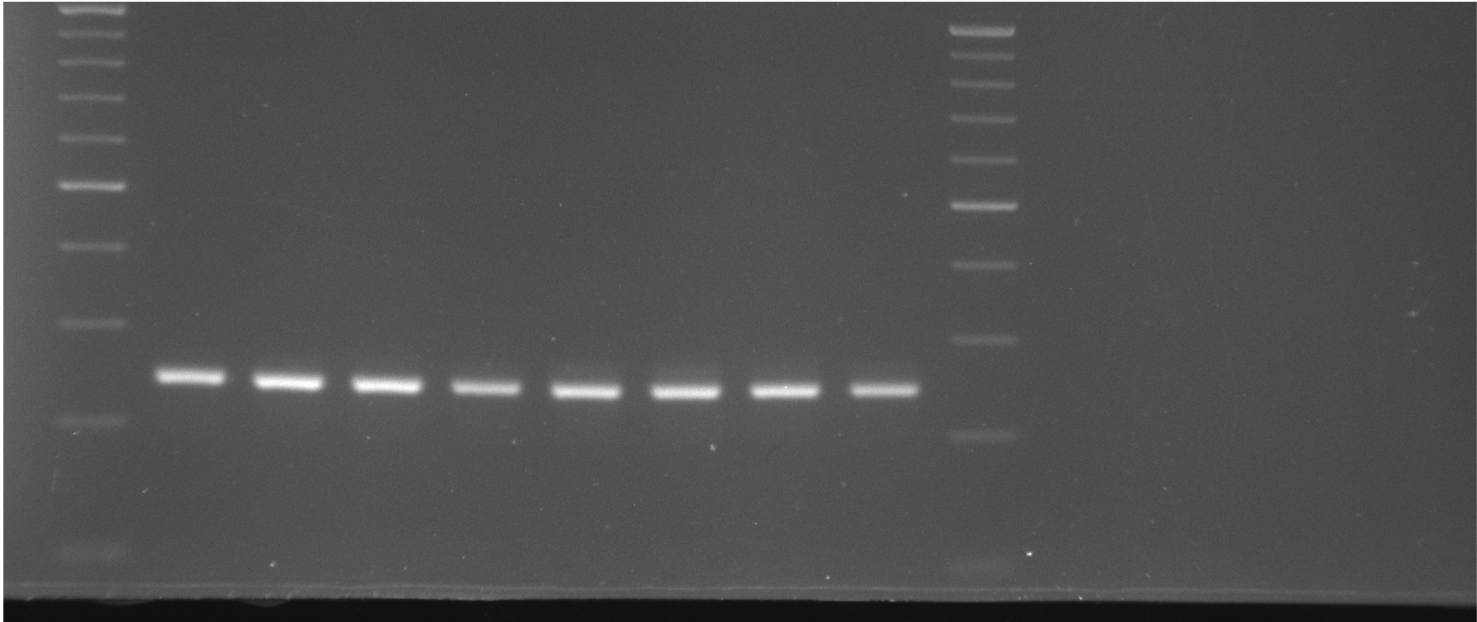

Supplement: Source data 1. [file elife-80683-data1.zip › Raw Data Gel Images/Suppl_Fig_4F-source-data-1.png]

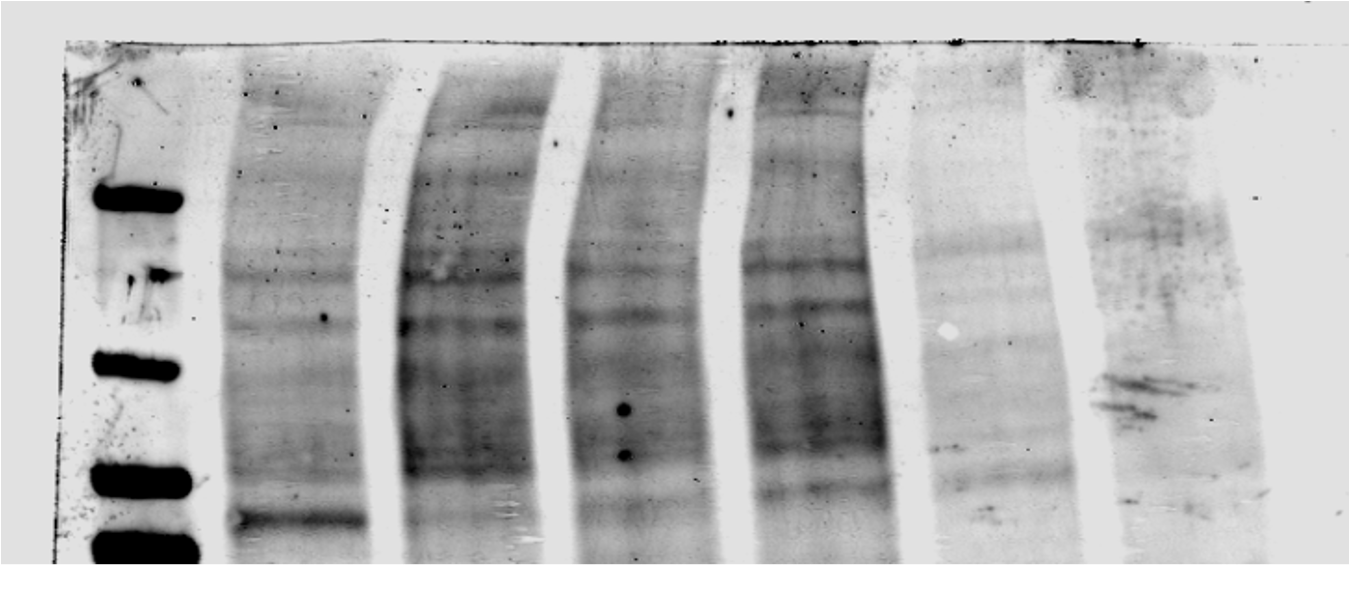

Supplement: Source data 1. [file elife-80683-data1.zip › Raw Data Gel Images/Suppl_Fig_4G-source-data-1.png]

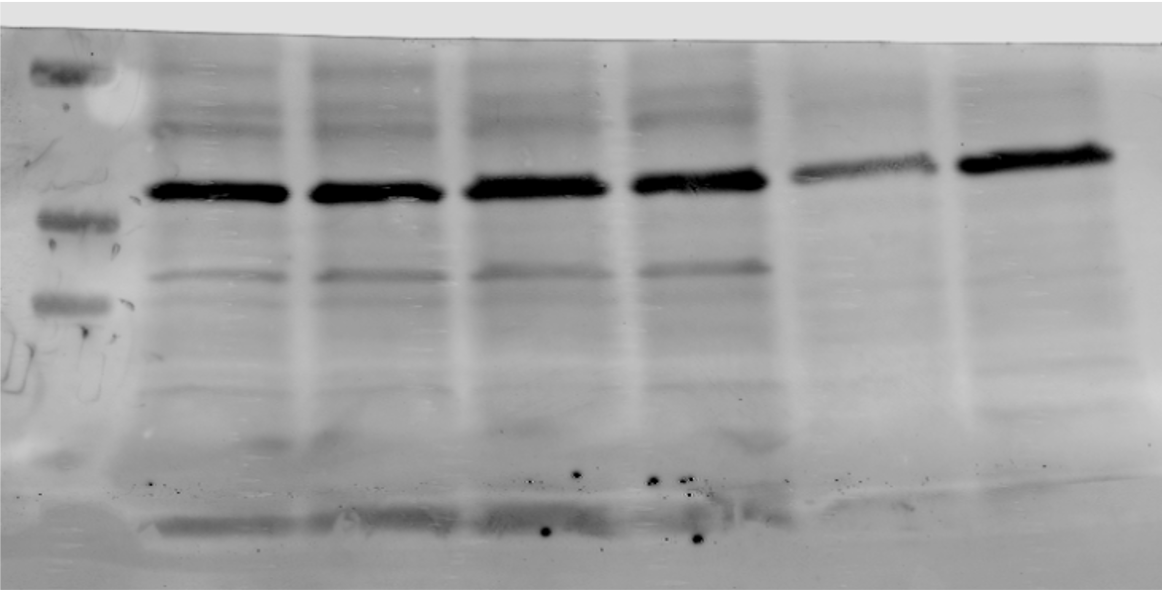

Supplement: Source data 1. [file elife-80683-data1.zip › Raw Data Gel Images/Suppl_Fig_4G-source-data-2.png]
